# Fabrication of Cross-Linked PMMA/SnO_2_ Nanocomposites for Highly Efficient Removal of Chromium (III) from Wastewater

**DOI:** 10.3390/polym14102101

**Published:** 2022-05-21

**Authors:** Nazeeha S. Alkayal

**Affiliations:** Chemistry Department, Faculty of Science, King Abdulaziz University, P.O. Box 80203, Jeddah 21589, Saudi Arabia; nalkayal@kau.edu.sa

**Keywords:** cross-linked PMMA, SnO_2_, nanocomposite, adsorption, Cr (III) removal

## Abstract

In recent times, developments in polymer application properties have required the design of different polymer structures more than ever. Cross-linked polymers (CPs) could be considered a good candidate material for potential applications when used in conjunction with nanoparticles. Cross-linked polymethyl methacrylate nanocomposites are considered to be one of the most commonly polymeric adsorbents due to their varied and simple modification methods. A new class of C-PMMA/SnO_2(a–d)_ nanocomposites have been fabricated as surface-selective adsorbents of Cr (III) with a good yield and different loading of SnO_2_ nanoparticles. The morphology, molecular structures, and thermal stability of the new cross-linked polymers were examined using a Scanning electron microscope (SEM), the Fourier Transform Infrared method (FTIR), X-ray diffraction (XRD), and Thermogravimetric Analysis (TGA). The adsorption study of C-PMMA/SnO_2_ was investigated, and an efficient level of adsorption for Cr (III) cations was detected. To evaluate the potential for the new polymers to be used as adsorbents against Cr (III) ions, the contact time, the initial concentration of Cr (III), and the effects of pH were studied. The introduction of SnO_2_ into the polymer network enhanced the efficiency of the adsorption of heavy metals. The C-PMMA/SnO_2_ is highly efficient at removing Cr (III) ions in wastewater samples at pH 6 for one hour. The adsorption study demonstrated that the adsorption capacity of C-PMMA/SnO_2_c for Cr (III) was 1.76 mg /g, and its adsorption isotherm agreed with the Langmuir adsorption model.

## 1. Introduction

Water pollution is becoming an increasingly important environmental issue as populations and industries continue to grow. Heavy metal ions are the most dangerous pollutants in wastewater because they are not biodegradable and can be collected through the food chain inside of organisms, causing many diseases and severe environmental pollution. As a result, any risk at ecosystem can cause damage to other organisms as well [1]. Among the heavy metals, chromium is considered one of toxic metals in the environment due to its flexible oxidizing properties. Chromium has two oxidizing states, chromium (III) and chromium (VI), and chromium (VI) is known to have carcinogenic properties, thus rendering it more toxic than chromium (III). However, in the presence of oxidizing impurities, Cr (III) can certainly be oxidized to chromium (VI) [2,3]. The primary industrial sources of chromium heavy metals are the leather industry, tanning industry, electroplating industry, textile industry, etc. Chromium must be significantly removed from wastewater before it is released into the environment or before it needs to be reformed [4,5]. Therefore, many approaches have reportedly developed more efficient technologies for removing chromium from water. For example, chemical precipitation, liquid extraction, ion exchange, precipitation, adsorption, membrane purifiers, reverse osmosis, and packing are all representative of such techniques and technologies [6,7,8,9,10].

However, adsorption is one of the safest and most cost-effective, and some direct methods can be used very efficiently to remove these metals from wastewater. The efficiency of adsorption depends mainly on the nature of the adsorbents. Many materials have been used as adsorbents, categorized into synthetic, natural adsorbents, and nanoscale materials such as activated carbon, synthetic zeolites, and diatomaceous earth [11,12,13,14]. However, some adsorbents suffer from a long equilibration time and a low capacity; therefore, preparing large volumes of material in a shorter period is challenging for water treatment. The polymer can create active motifs in the chains, increasing the chances for higher uptake so that it is considered a good candidate for adsorption. Several studies have been carried out using polymers such as PMMA as adsorbents to remove heavy metals from industrial wastewater because PMMA is stable, cheap, harmless, and a good material for use in water treatment. [15,16,17].

In the past decade, nanotechnology has been frequently applied successfully in various fields of science. However, recently its use to address heavy metal pollution in water and has become a rapidly growing and exciting field for environmentalists. The dispersion of various organic or inorganic particles in a polymer matrix has received significant attention due to more accessible and cheaper techniques for changing the properties of composites depending on the application required. Numerous nanofillers such as nanofibers, platelets, spheres, and nanotubes have been used mainly as reinforcements for polymer composites. The benefits of including other nanoparticles (NPs) into polymers such as PMMA have also been reported concerning heavy metal removal efficiency [18,19,20,21,22,23]. SnO_2_ is attracting a great deal of attention because it has a high conductivity, transparency, and sensitivity to gases. SnO_2_ is considered an n-type semiconductor providing many technical applications, operating as organic oxidation catalysts, solid-gas sensors, and environmental applications for the removal of dyes and heavy metal ions [24,25]. (SnO_2_) nanoparticles (SnO_2_ NPs) are highly adsorbent and have an active group surface, intense interactions with adsorbed materials either physical or chemical, a high mechanical stability and thermal properties [26] and is considered a suitable cation exchanger [27]. The use of SnO_2_ in polymers is a practical strategy for developing water purification methods. The presence of nanomaterials in the polymeric network gives these materials more flexibility and adsorption immobilization, which prevents nanoparticle recovery after water treatment [28]. As an example of the incorporation of SnO_2_ NPs into PMMA and employing them for water treatment, *m*-phenylenediamine polymethylmethacrylate/tin(IV)oxide (*m*-PMMA/SnO_2_) was applied for the degradation of methylene blue (MB) dye under UV light with an efficiency of 99% for 120 min [29]. Another instance is graphene-SnO_2_-PMMA which was utlized to decompose MB dye by different irradation levels [30].

In this work, the designed PMMA nanocomposite was prepared through a simple and low-cost method that proved to be a good approach to water treatment. This study broadened our understanding of how to create cross-linked polymethyl methacrylate (PMMA) nanocomposites and found new ways to apply them in water purification. This study opened a new route for developing high performance cross-linked PMMA nanocomposite for water treatment. The cross-linked PMMA/SnO_2_ nanocomposite was fabricated and used to remove Cr (III) ions. The prepared cross-linked polymers were characterized using FTIR, SEM, XRD, and TGA. C-PMMA/SnO_2_ nanocomposites were examined as an effective sorbent to remove chromium (III) from an aqueous solution. The pH effect, contact time, concentration of metal ions, and isothermal kinetics were investigated in the adsorption procedure.

## 2. Experiment

### 2.1. Materials

Poly (methyl methacrylate) (PMMA *M*_wt_ = 300 K) was obtained from Alfa Aesar by thermos Fisher, Erlenbachweg, Germany, Tin (IV) oxide nanoparticles were purchased from Nano Gate CO, Cairo, Egypt. (SnO_2_ < 100 nm). Tetrahydrofuran (THF; 99.5%) was purchased from Fisher Chemical, Loughborough, UK, and *p*-phenylenediamine (*p*-PDA) (C_6_H_4_NH_2_)_2_; Fluka, Loughborough, UK, and the chemicals were used without purification or treatment. Cr(NO_3_)_3_·9H_2_O (97%) was provided by Panreac, Milano, Italy. HCl (35%) supplied from LOBA Chemie, Mumbai, Indiaand used without any further purifications. ICP multi-element standard solution and yttrium solution were purchased from Merck, Darmstadt, Germany.

### 2.2. Instrumental

Thermo-gravimetric analysis (TGA) was performed on a TGA4724 from 0.00 to 77.50 min with heat flow E Indium: 191.5376 1/mW, dE relative: −23.3291 + 0.0000*T + 0.0000*T^2 and air flow rate of 10 °C /min. Fourier transform infrared spectra (FTIR) with a Nicolet Magna 6700 FT spectrometer were conducted at a transmittance of 100% with wavenumbers range (4000–600 cm^−1^) and scanning the rate was 32 scans per minute. X-ray diffraction (XRD) patterns were examined using a Bruker D8 Advance with Cu Kα radiation (wavelength 1.5418 Å) at a power of 40 kV and 40 mA, step size:0.02, using LynxEye one dimensional detector and 2theta range:10–80 degree at time/step:1.0 s. Scanning electron microscopy (SEM) imaging was performed with an FEI TENEO VS microscope equipped with an EDAX detector. The polymer sample was mounted on an aluminium stub using adhesive carbon tape and sputter-coated with 3 nm Iridium to avoid sample charging during imaging. Inductively Coupled Plasma Optical Emission Spectrometry (ICP-OES) measurements were used in the model of a Perkin Elmer ICP-OES model Optima 4100 DV, USA. An ICP-OES spectrometer was used with the following parameters: FR power, 1300 kW; frequency, 27.12 MHz; glass spray chamber according to Scott (Ryton), sample pump flow rate, integration time, 3 s; replicates, 3; 1.5 mL/min; wavelength range of monochromator 165–460 nm. Calibration curves were carried out using ICP multi-element standard solution and yttrium solution was added in as an internal standard.

### 2.3. Synthesis of C-PMMA/SnO_2_ Nanocomposites

C-PMMA/SnO_2_ nanocomposites were prepared using polycondensation as described in reference [31]. Briefly, 1 g of PMMA was dissolved in 50 mL THF solvent, then SnO_2_ nanoparticles with different ratios (5, 10, 20, and 40 wt%) were dispersed throughout the PMMA solution in the ultrasonic for 10 min. Then, *p*-PDA was added as a cross-linker, and the mixture was refluxed for 6 hours at 70 °C with regular shaking. The mixture was then placed into Petri dishes and left to dry for 24 h at 25 °C, and the films were collected for characterization and water treatment.

### 2.4. Adsorption Study

A standard solution of 1000 ppm of Cr (III) was prepared in deionized water. The batch methods were utilized for pure C-PMMA and C-PMMA/SnO_2(a–d)_. The standard solution of 5 ppm of Cr (III) was prepared and adjusted to pH values ranging from 2.0 to 7.0 with appropriate buffer solutions. The effect of pH on the selectivity of the adsorption of these materials for Cr (III) was studied. Regarding the study of the adsorption capacity of Cr (III) under batch conditions, standard solutions of 0–100 mg/L Cr (III) were prepared and adjusted to the optimum pH value of 6.0 and individually mixed with 20 mg C-PMMA/SnO_2_c using a mechanical shaker.

## 3. Results and Discussion

### 3.1. Structural Investigation

**Figure 1:**
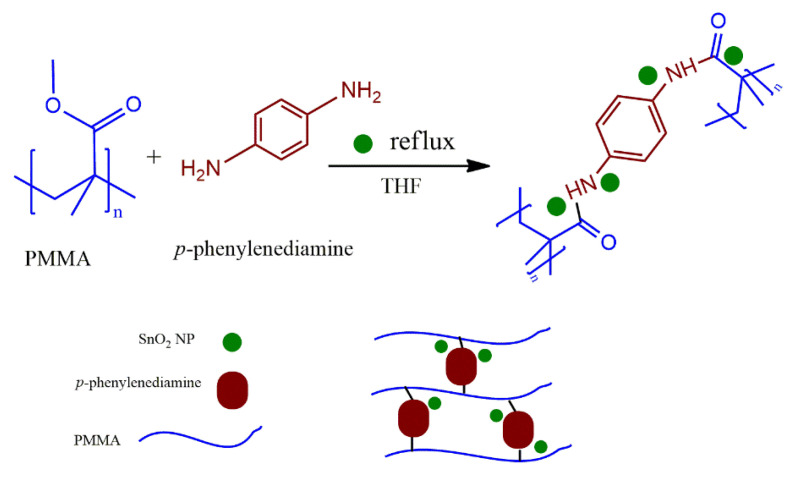
Representation diagram for synthesis of C-PMMA/SnO_2_ nanocomposites.

Cross-linked-PMMA/SnO_2(a–d)_ nanocomposites were prepared using an in-situ polymerization with different loadings of SnO_2_ NPs. The suggested abbreviations for C-PMMA/SnO_2(a–d)_ nanocomposites of several ratios of SnO_2_ NPs are listed in Table 1, and the schematic diagram for the nanocomposite’s fabrication is illustrated in Figure 1.

**Figure 2:**
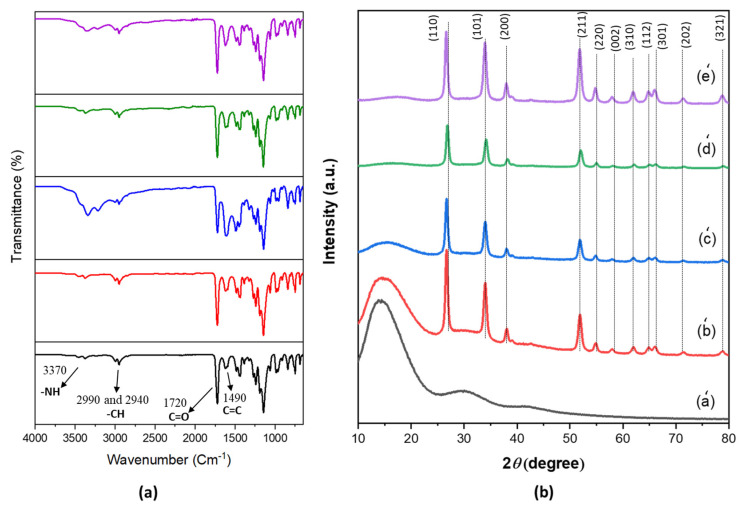
(**a**) FTIR spectra for pure C-PMMA and the C-PMMA/SnO_2(a–d)_ nanocomposites, (**b**) XRD diffraction patterns of (a’) C-PMMA, (b’) C-PMMA/SnO_2a_ (c’) C-PMMA/SnO_2b_, (d’) C-PMMA/SnO_2c_, and (e’) C-PMMA/SnO_2d_.

The FTIR spectra of the C-PMMA and the C-PMMA/SnO_2(a–d)_ nanocomposites are shown in Figure 2a. In the spectrum of C-PMMA, the peaks around 2990 and 2940 cm^−1^ are assigned to the CH stretching vibrations of aromatic and aliphatic compounds, respectively. A sharp peak appeared at 1720 cm^−1^, revealing the stretching vibrations of the carbonyl group C=O. The C=C stretching vibration of the aromatic rings has a peak around 1490 cm^−1^, and the peaks in the absorption range from 3450 to 3370 cm^−1^ are related to the stretching vibration of N–H. Additionally, the N–H bending has a peak at 1615 cm^−1^, which confirms the interaction between the carbonyl group of PMMA with the amine group of *p*-phenylenediamine in the cross-linking structure [29,31,32].

As shown in Figure 2b, the XRD diffractograms of C-PMMA/SnO_2(a–d)_ nanocomposites and pure C-PMMA were analyzed at the 2*θ* range from 10° to 80°. The XRD diffractogram (a) of pure C-PMMA in Figure 2b demonstrated the broadened peaks around 15°, 30°, and 43°, which were attributable to the amorphous nature of C-PMMA. After adding SnO_2_ NPs to the C-PMMA, the polymeric matrix has crystalline peaks that become sharper, and the intensity also increases from (b) to (e), caused by the rising of the intermolecular interaction as presented in Figure 2b. The XRD patterns (b)–(e) display the broad peaks related to the C-PMMA at 15°, 30°, and 43°, and there are other sharp peaks assigned to SnO_2_ NPs that confirm the structure of the nanocomposite polymers. The XRD studies show that the addition of SnO_2_ to C-PMMA yielded a considerable increase in the peak intensity compared to that of the pure C-PMMA, indicating an increase in the crystallinity. The crystallinity of the polymer nanocomposite can be determined by calculating the area under the peak and normalized based on the peak area of the pure polymer. As a result, the crystallinity of the obtained polymer increased withincreasing the content of SnO_2_ NPs in the nanocomposite.

### 3.2. Morphological Investigation

**Figure 3:**
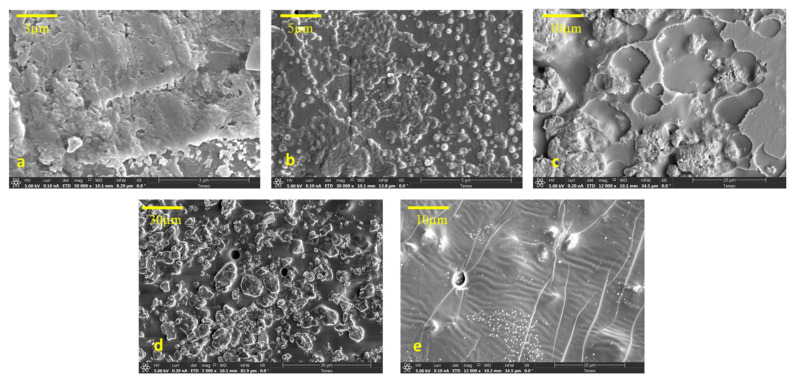
SEM images for (**a**) C-PMMA (50,000×), (**b**) C-PMMA/SnO_2a_ (30,000×), (**c**) C-PMMA/SnO_2b_ (12,000×), (**d**) C-PMMA/SnO_2c_ (5000×), and (**e**) C-PMMA/SnO_2d_ (12,000×).

As shown in Figure 3, the (SEM) images illustrate the surface morphologies of the C-PMMA and C-PMMA/SnO_2(a–d)_ nanocomposites. In Figure 3a, the SEM image of C-PMMA shows smoothed holes on the polymer’s surface upon magnification (x = 50,000). The SEM images of Figure 3b–e are assigned to C-PMMA/SnO_2(a–d)_ nanocomposites after the addition of SnO_2_ NPs. According to the excellent interaction between SnO_2_ and C-PMMA, the surface morphology changed and rounded to the micropores. As a result, there is an apparent deformation of the circular hole when a load of SnO_2_ increases.

By using the EDX model, the existence of SnO_2_ NPs in C-PMMA/SnO_2_ was studied. Figure S1a shows three signals of C, O, and N in C-PMMA, while the EDX spectra in Figure S1b–e demonstrate the exhibited peaks corresponding to the C, N, O, and Sn elements. As a result, when the ratio of SnO_2_ NPs in the polymeric matrix increases, the composition of Sn elements increases.

### 3.3. The Thermal Stability by TGA

**Figure 4:**
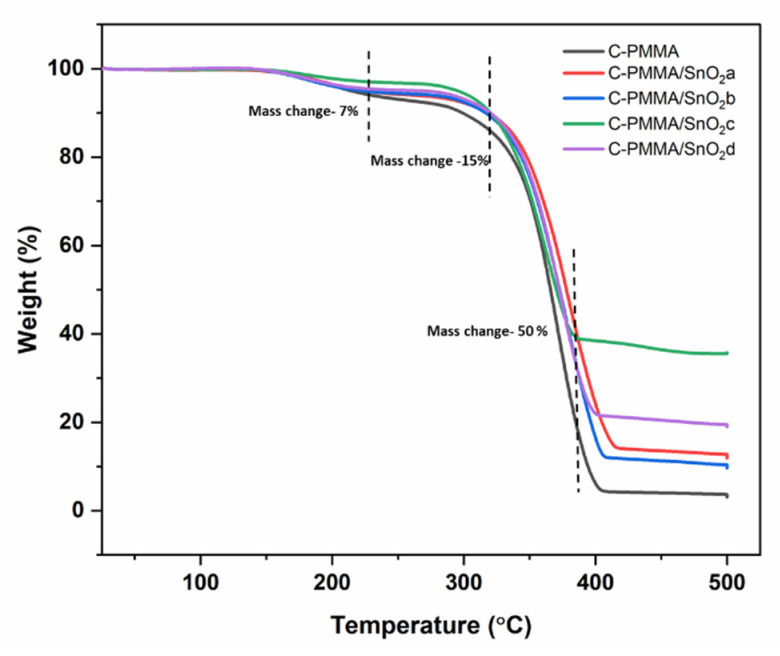
TGA of C-PMMA and C-PMMA/SnO_2(a–d)_.

Tests of the thermal stability of C-PMMA and C-PMMA/SnO_2(a–d)_ nanocomposites were performed using TGA under air atmosphere conditions with a heating rate of 10°C/min and a temperature range of 25–500 °C as shown in Figure 4. The weight loss of C-PMMA occurred in a three-step process and the starting temperature of weight loss occurred at 175 °C, which indicates that the polymer can remain stable at this temperature, but become unstable at more than 175 °C [29,31,32]. In the thermal analysis, a similar behavior of C-PMMA is detected for C-PMMA/SnO_2(a__–__b)_ with the difference being that the initial temperature of the mass loss happened at 240 °C instead. The first step of weight loss happened in the temperature range of 175–235 °C with a weight loss of 6%, and this corresponds to the removal of water. The second and third stages of weight loss occurred in the temperature ranges of 260–360 and 370–433 °C, respectively. The second stage of weight loss, with the amount of 20% that is related to the degradation of the cross-linking bond and the polymer chain, was totally burned and decomposed in the third stage of weight loss with an amount above of 60%. The polymer is destroyed above 500 °C; therefore, it can be concluded that the stable temperature rises to 240 °C due to the incorporation of SnO_2_ NPs into the polymer. The stable temperature of the polymer will increase about 60 °C due to the anchoring of NPs into the polymer matrix. The temperature of the decomposition percentages for the mass losses of 10%, 25%, and 50% were listed in Table 2. As a result, the T_10_, T_25_, and T_50_ of the C-PMMA/SnO_2_ nanocomposites are higher than for pure C-PMMA.

### 3.4. Adsorption Studies

**Figure 5:**
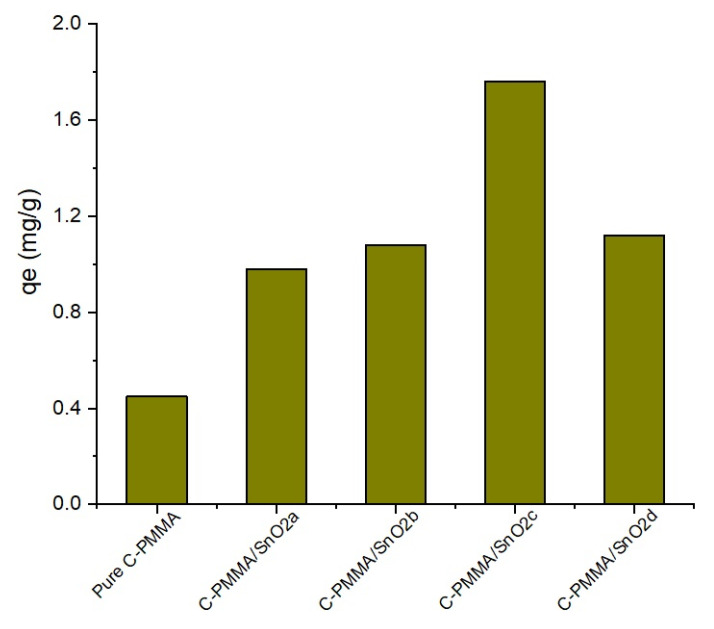
Surface selectivity study of pure C-PMMA and C-PMMA/SnO_2(a–d)_ for Cr (III).

Table 3 demonstrates the adsorption capacities (mg/g) of pure C-PMMA and C-PMMA/SnO_2(a–d)_ for chromium (III) cations, and it was found that the C-PMMA/SnO_2(a–d)_ has an excellent uptake of Cr (III) compared to pure C-PMMA. Figure 5 indicates that C-PMMA/SnO_2c_ has the highest adsorption capacity of Cr (III). As a result, the chromium (III) cations and C-PMMA/SnO_2c_ were chosen for further adsorption experiments. The adsorption capacity q_e_ (mg/g) and the distribution adsorption coefficient (K_d_) were calculated according to the following equation

(1) $$q_{e}=\frac{\;\left(C_{i}-C_{f}\right)V}{m}$$

(2) $$K_{d}=\frac{\;\left(C_{i}-C_{f}\right)}{C_{f}}\times \frac{V}{m}\;$$

where the V is the volume (L) of the solution, the amount of adsorbent is represented as m (g), C_i_ is the initial concentration of the metal cations (mg/L), and C_f_ is the final concentration of the metal cations (mg/L). The effect of the adsorbent’s efficiency to remove the heavy metals was studied using different factors such as pH value, contact time, and metal concentration. The experiments were repeated in three batches and the data are the values of three measurements in all the figures.

#### 3.4.1. Effect of pH

**Figure 6:**
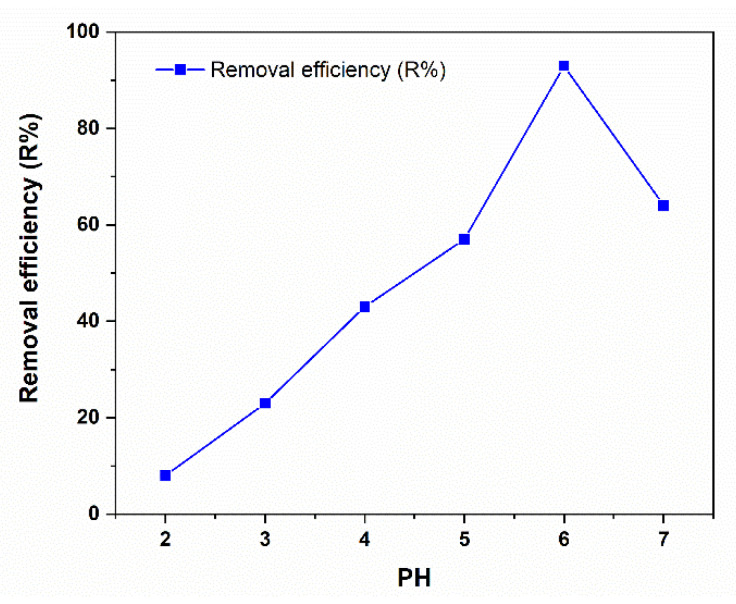
The effect of pH on the adsorption of Cr (III) (5 mg/L) on 20 mg of C-PMMA/SnO_2_c at 25 °C.

Figure 6 illustrates the effect of pH on the removal efficiency (R%) of Cr (III). As the pH increases, the adsorption capabilities of Cr (III) increase significantly. The results indicate that the significant adsorption of Cr (III) occurred at pH = 6, which was then employed in subsequent adsorption studies. In an acidic medium, high concentrations of H^+^ cooperate with the surface, which decreases the adsorption at the surface. At high a pH, the same occurrence can be used to describe the low adsorption efficiency because the hydroxyl groups can reduce the removal of chromium.

(3) $$R\%=\frac{C_{i}-C_{f}}{C_{i}}\times 100$$

#### 3.4.2. Effect of Contact Time

**Figure 7:**
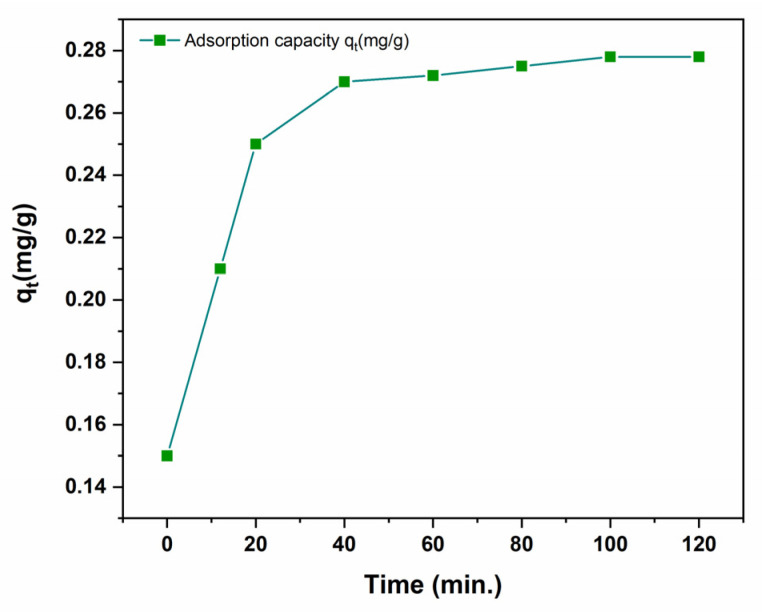
The impact of contact time on adsorption of Cr (III) 20 mg of C-PMMA/SnO_2_c at pH 6.0 and 25 °C.

Figure 7 displays the effect of contact time on the removal of Cr (III) using C-PMMA/SnO_2c_ nanocomposite. The adsorption of Cr (III) was fast in the 20 min interval which is attributable to the high availability of the Cr (III) ions to the ion-dipole sites of the C-PMMA/SnO_2c_. Thus, the adsorption rate achieved equilibrium within 40 min.

#### 3.4.3. Impact of Initial Concentration of Cr (III)

**Figure 8:**
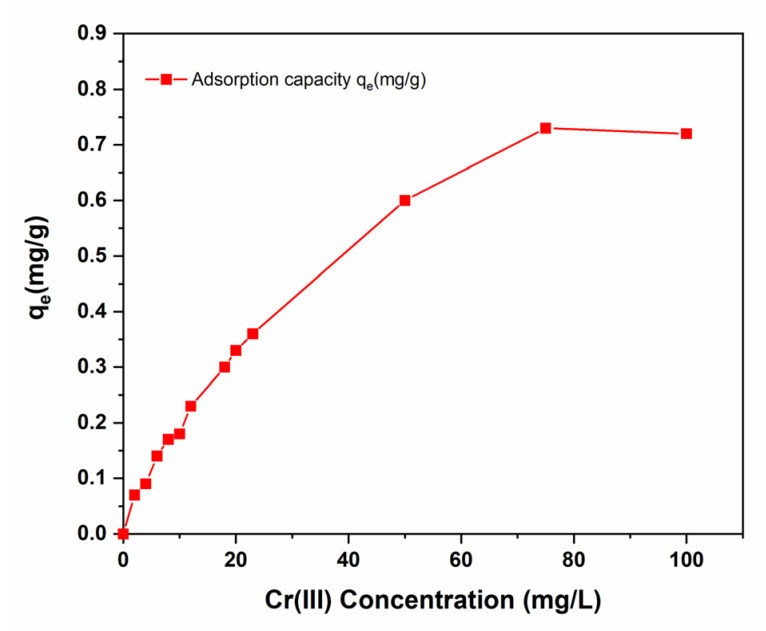
The of Cr (III) adsorption profile related to different concentration on 20 mg of C-PMMA/SnO_2c_ at pH 6.0 and 25 °C.

The effect of the initial concentration of Cr (III) on the adsorption efficiency of C-PMMA/SnO_2_c was examined in the range from 0–100 mg/L as presented in Figure 8. As the amount of Cr (III) cations in the solution increases, the adsorption capacity of C-PMMA/SnO_2c_ increases to reach a maximum value of q_e_ = 75 mg/g at 80 mg/L. Thus, the 20 mg/L was chosen for optimum adsorption conditions to study the adsorption capacity of C-PMMA/SnO_2c_ at low concentrations.

### 3.5. Adsorption Isotherms

**Figure 9:**
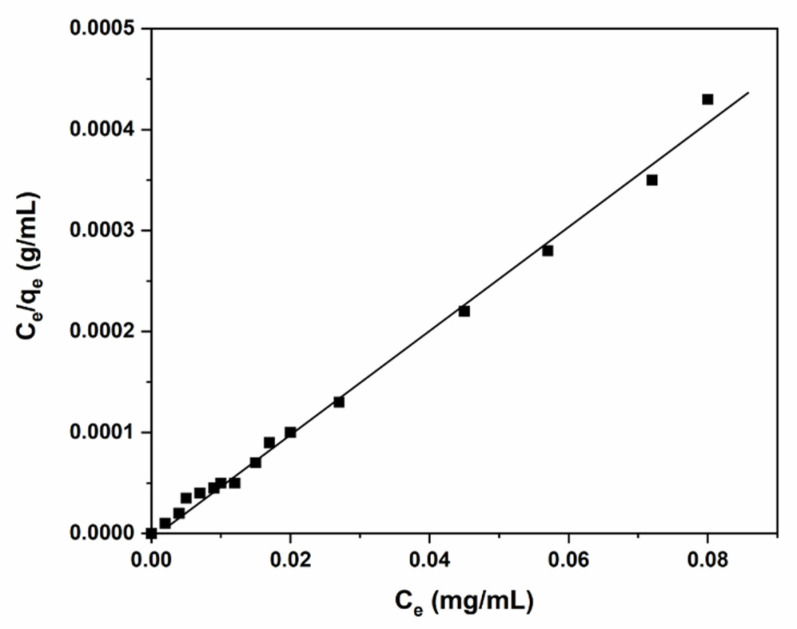
Isotherm model of the adsorption Cr (III) on 20 mg of C-PMMA/SnO_2c_ at pH 6.0, 25°C and at different concentrations (0–100 mg/L) of Cr (III).

The interaction between adsorbent and adsorbate was investigated using the adsorption isotherm model. In this study, the adsorption isotherm plays an essential role in predicting the analytical results of the Langmuir model. [33] Figure 9 indicates that the results align with the Langmuir equation. The validity of the Langmuir isotherm equation is confirmed for the data obtained through the linear plot; it is adsorbed to the Langmuir adsorption isotherm model.

The Langmuir isotherm measured the homogeneity of the surface area sites using the classical adsorption isotherm, as shown in the equation below

(4) $$\frac{C_{e}}{q_{e}}=\frac{C_{e}}{Q_{m}}+\frac{1}{{\mathrm{bQ}}_{m}}$$

where C_e_ (mg/L) and q_e_ (mg/g) are Cr (III) concentration and the adsorption capacity of the polymer, respectively. Q_m_ (mg/g) is the maximum adsorption capacity of Cr (III) and b (L/mg) is the Langmuir constant. Langmuir constants Q_m_ and b can be calculated from a linear plot of C_e_/q_e_ against Ce with a slope and intercept equal to 1/Q_m_ and 1/Q_m_b, respectively. In addition, the basic properties of the isotherm adsorption model can be attained under the conditions of a constant dimensional separation factor or equilibrium parameter, R_L,_ as follows

(5) $$R_{L}=\frac{1}{1+K_{L}\;*\;C_{o}}$$

C_o_ = initial Cr (III) concentration (mg/g) and K_L_ = constant of Langmuir. As listed in Table 4, the R_L_ value of Cr (III) adsorption on C-PMMA/SnO_2_c is 0.03, demonstrating a highly desirable adsorption. As reported in [34,35], the nature of the adsorption isotherm can be determined by the value of R_L_, and an obtained value between 0 < R_L_ < 1 signals good adsorption. In the adsorption process of Cr (III) for C-PMMA/SnO_2c_, Langmuir constants Q_o_ and b were 72.32 mg/g and 0.24 L/mg, respectively. The correlation coefficient (R^2^) obtained from the Langmuir model was calculated as 0.98. The data show that attaining the Langmuir model and C-PMMA/SnO_2_c has a monolayered and homogeneous surface during the adsorption process.

The new adsorbent in this study and another different adsorbent were listed in Table 5. As a result, it was found that the present adsorbent has a good adsorption efficiency toward Cr (III) with less dosage and short time, and it can be successfully applied for removal Cr (III) from wastewater.

## 4. Conclusions

The prepared cross-linked PMMA/SnO_2_ had a network structure that was beneficial for the exposure of adsorptive sites on the cross-linked polymer adsorbent towards the adsorbate. In this work, new cross-linked PMMA nanocomposites were synthesized and applied for adsorption applications. The adsorption study results show that the obtained nanocomposites have a higher adsorption efficiency than the pure cross-linked polymers because the presence of SnO_2_ NPs enhances the ability of adsorption. The maximum adsorption efficiency was found at 60 min in the pH 6 range. The adsorption isotherm model showed that the Langmuir isotherm model was the best to describe the metal adsorption process. This study broadened our understanding of how to create cross-linked polymethyl methacrylate (PMMA) nanocomposites and found new ways to apply them in adsorption. This study opened a new route for developing high-performance cross-linked polymer adsorbents for water purification. The findings of the present study may have important implications for different applications.

## Figures and Tables

**Table 1:** Chemical composition of C-PMMA and C-PMMA/SnO2(a–d) nanocomposites.

Abbreviation	PMMA%	*p*-Phenylenediamine (*p*-PDA) %	SnO_2_ wt.%
**C-PMMA**	80	20	-
**C-PMMA/SnO_2a_**	80	20	5
**C-PMMA/SnO_2b_**	80	20	10
**C-PMMA/SnO_2c_**	80	20	20
**C-PMMA/SnO_2d_**	80	20	40

**Table 2:** Thermal behavior of C-PMMA and C-PMMA/SnO_2(a–d)_ nanocomposites.

Sample	Temperature (°C) for Various Percentage Decompositions		
	T_10_	T_25_	T_50_
**C-PMMA**	295	384	410
**C-PMMA/SnO_2a_**	352	396	424
**C-PMMA/SnO_2b_**	351	392	416
**C-PMMA/SnO_2c_**	354	386	415
**C-PMMA/SnO_2d_**	315	392	417

**Table 3:** The adsorption studies of pure C-PMMA and C-PMMA/SnO_2(a–d)_ for chromium (III) ions at 25 °C.

Code	Selected Ions	q_e_ (mg/g)	K_d_ (mL/g)
**C-PMMA**	Cr (III)	0.45	968.42
**C-PMMA/SnO_2a_**	Cr (III)	0.98	1674.92
**C-PMMA/SnO_2b_**	Cr (III)	1.08	2749.65
**C-PMMA/SnO_2C_**	Cr (III)	1.76	43518.24
**C-PMMA/SnO_2d_**	Cr (III)	1.12	5673.62

**Table 4:** Langmuir isotherm constants of C-PMMA/SnO_2_c toward the adsorption of Cr (III), at pH 6.0 and 25 °C.

Material	Q_o__(_mg/g)	b (L/mg)	R^2^	R_L_
C-PMMA/SnO_2_c	72.32	0.24	0.98	0.03

**Table 5:** Comparison of the adsorption efficiency of C-PMMA/SnO_2_ and the adsorption efficiencies of various adsorbents for Cr (III).

Adsorbent	R%	PH	Dose	Time	Ref.
**Aluminum oxide hydroxid**	90%	4	1 g	60 min	[36]
Modified carbon nanotubes (M-CNTs)	18%	7	150 mg	120 min	[6]
cross-linked acidic tetrapolymer (CPZA)	90%	5.5	30 mg	60 min	[37]
Eggshell powdered marble	40% 25%	5 5	20 mg 12 mg	14 h 30 min	[38] [38]
sugarcane pulp residue	90%	6	0.5 g	24 h	[39]
C-PMMA/SnO_2_	90%	6	20 mg	40 min	This work

## Data Availability

Not applicable.

## Supplementary Materials

The following supporting information can be downloaded at: https://www.mdpi.com/article/10.3390/polym14102101/s1, Figure S1: EDX analysis for (a) C-PMMA, (b) C-PMMA/SnO_2a_, (c) C-PMMA/SnO_2b_, (d) C-PMMA/SnO_2c_, and (e) C-PMMA/SnO_2d_.
