# Supplementary material for: Fabrication of Cross-Linked PMMA/SnO2 Nanocomposites for Highly Efficient Removal of Chromium (III) from Wastewater
Source: Polymers (Basel). 2022 May 21;14(10):2101. doi: 10.3390/polym14102101 (PMC9145786; doi:10.3390/polym14102101)
Supplement: Supplementary file 1 [file polymers-14-02101-s001.zip › polymers-1712438-supplementary.pdf]

Fabrication of Cross-Linked PMMA/SnO_2 Nanocomposites for Highly Efficient Removal of Chromium (III) from Wastewater

Nazeeha S. Alkayal

Chemistry Department, Faculty of Science, King Abdulaziz University, P.O. Box 80203, Jeddah 21589, Saudi Arabia; nalkayal@kau.edu.sa

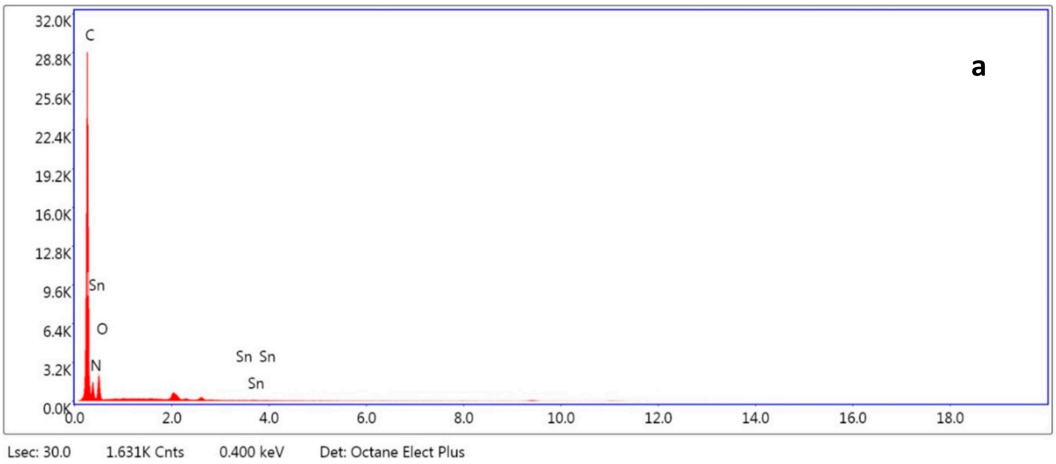

Element	Weight %	Atomic %
C K	61.93	66.71
N K	22.04	20.36
O K	16.00	12.93
SnL	0.03	0.00

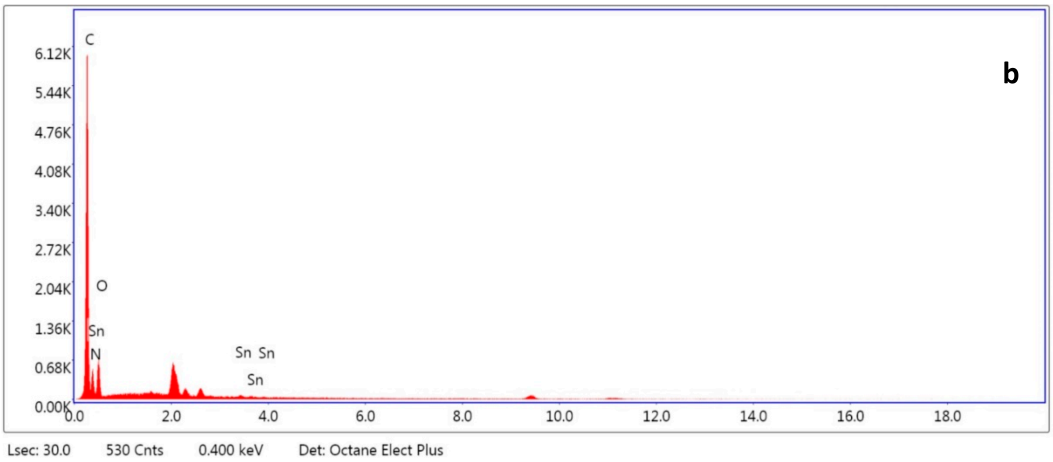

Element	Weight %	Atomic %
C K	68.14	72.98
N K	16.84	15.47
O K	14.25	11.46
SnL	0.77	0.08

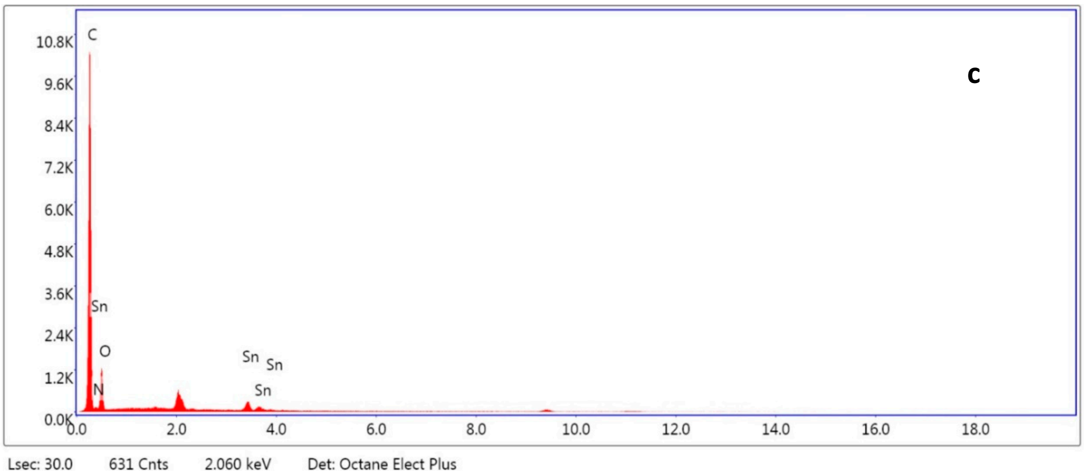

C K	75.43	82.87
N K	0.01	0.01
O K	20.18	16.64
SnL	4.38	0.49

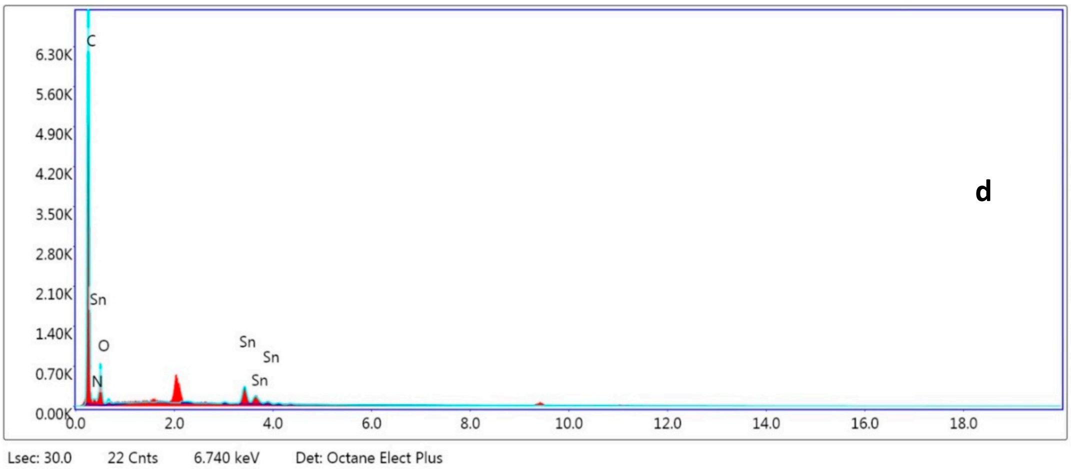

C K	74.90	85.34
N K	0.01	0.01
O K	15.88	13.58
SnL	9.20	1.06

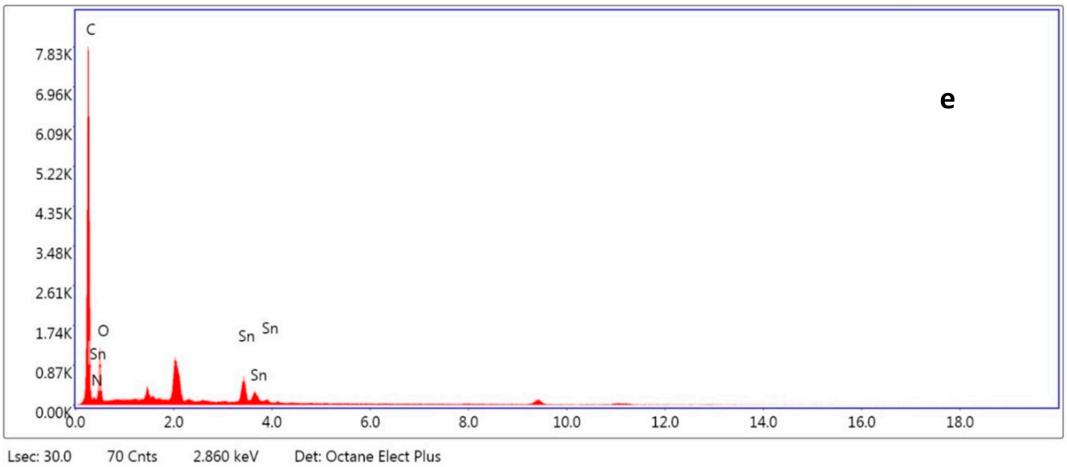

C K	70.92	82.84
N K	0.01	0.01
O K	18.08	15.85
SnL	10.99	1.30

Figure (SI) EDX analysis for (a) C-PMMA, (b) C-PMMA/SnO_{2a} , (c) C-PMMA/SnO_{2b}, (d) C-PMMA/SnO_{2c}, (e) C-PMMA/SnO_{2d}.
